# Impact of the SARS-CoV-2 pandemic on acute coronary syndrome patients admitted to an urban academic hospital in Soweto, South Africa

**DOI:** 10.11604/pamj.2024.47.160.37066

**Published:** 2024-04-03

**Authors:** Suzan Leon, Priya Parbhoo, Ruchika Meel

**Affiliations:** 1 Department of Internal Medicine, Faculty of Health Sciences, University of the Witwatersrand, Johannesburg, South Africa,; 2 Department of Internal Medicine, Division of Cardiology, Chris Hani Baragwanath Academic Hospital, University of the Witwatersrand, Johannesburg, South Africa

**Keywords:** Acute coronary syndrome, coronavirus disease 2019, ST-segment elevation myocardial infarction, non-ST-segment elevation myocardial infarction

## Abstract

**Introduction:**

recent worldwide data has shown a concerning decline in the number of acute coronary syndrome (ACS) related admissions and percutaneous coronary intervention (PCI) procedures during the coronavirus disease 2019 (COVID-19) pandemic. We suspected a similar trend at Chris Hani Baragwanath Hospital (CHBAH).

**Methods:**

a retrospective descriptive study was conducted to evaluate and compare all ACS-related admissions to the cardiac care unit (CCU) at CHBAH in the pre-COVID-19 (November 2019 to March 2020) and during COVID-19 periods (April 2020 to August 2020).

**Results:**

the study comprised 182 patients with a mean age of 57.9 ±10.9 years (22.5% females). Of these, 108 (59.32%) patients were admitted in the pre-COVID-19 period and 74 (40.66%) during COVID-19 (p=0.0109). During the pre-COVID-19 period, 42.9% of patients had ST-segment-elevation myocardial infarction (STEMI), 39.2% with non-ST-segment -elevation myocardial infarction (NSTEMI) and unstable angina (UA) was noted in 18.52%. In contrast, STEMI was noted in 50%, NSTEMI in 43.24% and UA in 6.76% of patients during the COVID-19 period. A statistically significant difference in STEMI and NSTEMI-related admissions was not noted, however, there was a greater number of admissions for UA during the pre-COVID-19 period (18.52% vs 6.76%, P =0.013). Only a third of the patients with STEMI received thrombolysis during the pre-and COVID-19 periods (30.4% vs 37.8%, P=0.47). No difference in the number of PCI procedures was noted between the pre-and during the COVID-19 periods (78.7% vs 72.9%, P=0.37).

**Conclusion:**

there was a difference in overall ACS admissions to the CCU between pre-and during COVID-19 periods, however no difference between STEMI and NSTEMI in both periods. A higher number of UA admissions was noted during the pre-COVID-19 period. During both periods, the use of thrombolysis was low for STEMI and no difference in PCI was noted.

## Introduction

The coronavirus disease 2019 (COVID-19) is a life-threatening respiratory infection caused by severe acute respiratory syndrome coronavirus 2 (SARS-CoV-2). The first case was reported in Wuhan China on 8^th^ December 2019 [1]. The rapid spread of the virus worldwide led to the declaration of COVID-19 as a pandemic by the World Health Organization (WHO) on March 11 2020, a mere 3 months from the index case [2]. To combat and contain the spread of the virus, many countries implemented stringent lockdown measures [3]. In South Africa, the first confirmed case of COVID-19 was reported by the Department of Health on 5^th^ March 2020, with the subsequent introduction of lockdown measures on 26^th^ March 2020 [4].

Reports around the globe have confirmed a significant reduction in the number of hospitalizations for patients with acute coronary syndrome (ACS) and those requiring percutaneous coronary intervention (PCI) during the pandemic [5]. Different datasets from countries within Europe [6-10], Asia [11-13], and North and Latin America [14-16], have demonstrated 30% to 50% reductions in ACS admissions and PCI procedures. The decrease in ACS admissions was associated with an increase in morbidity and mortality [6-9]. There is limited data of this nature emanating from Africa. In a survey conducted by the European Society of Cardiology (ESC), only 4.7% of responses confirming the above reductions originated from the African continent [5]. A report from a few private hospitals in South Africa has noted similar findings [17].

Data from Africa and South Africa is very scarce. Whilst statistics are available for a few private hospitals within the country [17], similar information from tertiary state hospitals is lacking. In a developing country such as South Africa with already strained public health care facilities and numerous socioeconomic challenges, we suspected a poor outcome of patients with ACS during the pandemic. We hypothesized that, in keeping with reports from global studies, there would be a significant decline in the number of ACS admissions to the CCU as well as PCI/coronary artery bypass grafting (CABG) procedures in the COVID-19 period at CHBAH.

**Aim:** the study aimed to analyze the impact of COVID-19 on ACS admissions to the cardiac care unit (CCU) in Chris Hani Baragwanath Academic Hospital (CHBAH), Africa's largest tertiary state hospital, located in Soweto on the outskirts of Johannesburg city.

**Objectives:** 1) to describe and compare the demographic and clinical characteristics of the patients admitted to CCU between pre-COVID-19 and during COVID-19 periods, 2) to determine the number of patients admitted to CCU with ACS and associated comorbidities between pre-COVID-19 and during COVID-19 periods, 3) to determine the complications and rate thereof between pre-COVID-19 and during COVID-19 periods and between the two periods 4) to compare the management of patients between pre-COVID-19 and during COVID-19 periods.

## Methods

**Figure 1:**
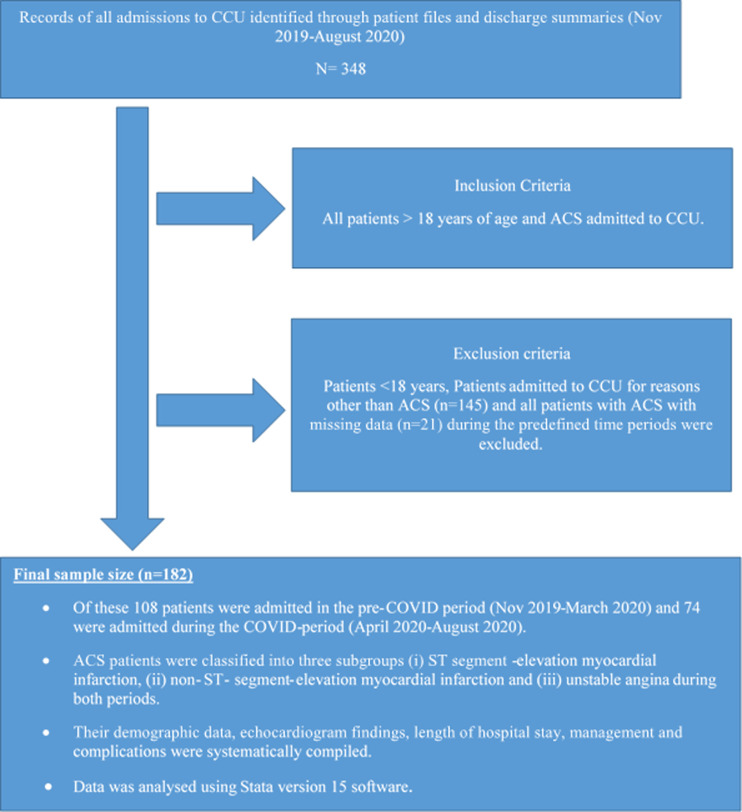
flow diagram depicting the methodology used in the study

**Study design and site:** this was a retrospective analytic cross-sectional study conducted at CHBAH in the pre-COVID-19 era (November 2019 to March 2020) and during the COVID-19 period (April 2020 to August 2020). All patients with ACS who were admitted to the CCU pre-COVID-19 era (November 2019 to March 2020) and during the COVID-19 period (April 2020 to August 2020) were considered for analysis. These patients would have had to have tested COVID-19 negative by PCR before admission to the CCU, due to the absence of isolation facilities. As a result, patients with COVID-19 and ACS were excluded from this study. Patients admitted to CCU for reasons other than ACS and all patients with missing data during the predefined time periods were excluded (Figure 1).

### Variables

***Outcomes:*** ACS was defined according to the Joint Task Force of the European Society of Cardiology, the American College of Cardiology Foundation, the American Heart Association, and the World Heart Federation (ESC/ACCF/AHA/WHF) fourth universal definition for myocardial infarction [18,19]. Data was analyzed for the three ACS groups: (i) ST-segment-elevation myocardial infarction (STEMI), (ii) non-ST-segment-elevation myocardial infarction (NSTEMI) and (iii) unstable angina (UA).

***Exposure:*** the two study periods were considered to be potential exposures in his study. We stratify our analysis based on whether the person was admitted pre-COVID-19 or during COVID-19.

***Predictors/risk factors:*** the most common risk factors evaluated in this study were hypertension, diabetes, smoking, having a family history of CAD, BMI and previous IHD. Major complications related to ACS were defined as heart failure with reduced ejection fraction (HFrEF), cardiogenic shock, arrhythmias, thromboembolism (left ventricular clot, critical limb ischemia and stroke), cardiac arrest, varying degrees of atrioventricular block, left ventricular aneurysms and in-hospital mortality.

***Potential confounders and effect modifiers:*** the possible confounders were age, gender, ethnicity, symptoms, length of hospital stay and medication.

**Data collection:** data was collected by the principal investigator by reviewing all discharge summaries and PCI reports of patients admitted to CCU. A consecutive convenience sampling method within the defined study period was used. The number of admissions, demographic data, echocardiogram findings, length of hospital stay, management and complications were obtained, and the corresponding data sheet was compiled.

**Bias:** since a record review was used, there is a possibility of information bias. This is due to missing information on some variables that can occur in the patient's records and the absence of some crucial information worth including in the study. Participant selection bias could have occurred in this study since we just included patients admitted at CHBAH who might not be a true representative of the overall picture of ACS admission in South Africa. This selection bias may affect the generalizability of our findings to other settings since a convenient sampling was used.

**Quantitative variables:** they were analysed using descriptive statistics and inferential statistics. Significance was set at 5%. P-values were used to assess statistical significance.

**Statistical analysis:** in the descriptive statistics phase of our analysis, categorical variables such as patients' socio-demographic and clinical characteristics, co-morbidities, complications, and mortality were summarized using frequencies and percentages. Continuous variables like age and length of hospital stay were summarised using mean(standard deviation (SD)) if the data was normally distributed and was median (inter-quartile range (IQR)) if the data was not normally distributed. Normality assumption was assessed using the Shapiro-Wilk test and p-values>0.05 indicate the variables were normally distributed.

To compare the different demographic and clinical variables of patients admitted to CCU pre-COVID-19 and during COVID-19, we used the Z-proportion test and Chi-square test for categorical variables. The independent T-test was used to compare normally distributed continuous variables between pre-COVID-19 and during COVID-19 while the Mann-Whitney test was used to compare non-normal continuous variables pre-COVID-19 and during COVID-19.

To determine the number of patients admitted to CCU with ACS and associated comorbidities during the COVID-19 period in comparison with the pre-COVID-19 period, we used the Z-proportion test and Chi-square test for categorical variables since the variables were categorical. To determine the complications and rate thereof between the two periods, we used the Z-proportion test and Chi-square test for categorical variables since the variables were also categorical. To compare the management of patients in the two periods, we used the Z-proportion test and Chi-square test for categorical variables since the variables were categorical. The Chi-square assessed independence or association between two categorical variables which was the case in our study. The Z-proportion test assessed two independent proportions as we used this for the admissions in the two study period.

For all hypothesis tests, a p-value of less than 0.05 was considered statistically significant. Stata version 17 was used for statistical analysis.

## Results

A total of 182 patients with ACS were admitted to the CCU during this study period. More admissions were observed during the pre-COVID period [59.34% (n=108)] compared to the COVID period [40.66% (n=74)]. There was a statistically significant difference between the number of admissions (P =0.0109) during the two periods.

**Demographic and clinical characteristics:** Table 1 summarizes the demographic and clinical characteristics of the cohort. In the overall group, males predominated. The number of males and females admitted in the two periods was similar, and therefore no significant difference in the admissions by gender was noted (P=0.905). Patients of the black race formed the majority of those admitted, as expected by the considerable proportion of black communities serviced by CHBAH. Pre-COVID-19 (48.07%) and COVID-19 (47.3%) numbers were similar for black patients. Although more Asian (31.3%) patients were seen in the pre-COVID and more Caucasians (37.84%) in the COVID-19 period, there was no difference in the admissions by ethnicity between the two groups (P=0.387). Beta-blockers, angiotensin-converting enzyme inhibitors, dual antiplatelet therapy and statins were the most prescribed drugs in both periods. Traditional cardiovascular risk factors and their prevalence in the pre-COVID-19 and COVID-19 groups are indicated in Table 1. Of note, there was a significantly lower number of patients with dyslipidaemia (P<0.001) and a family history of CAD (P<0.001) in the pre-COVID-19 group. Although the total length of hospital stay was separated by a median of 1 day between the groups, patients admitted during the COVID-19 period [4 (IQR 3-7)] stayed for a significantly longer duration (P =0.016) than those in the pre-COVID-19 period [3 (IQR 2-5)].

**Table 1:** a comparison between demographic and clinical characteristics of study patients between pre-COVID-19 and post COVID -19 periods

Variable	Categories	Pre-COVID-19 admission n=108 (%)	COVID-19 admission n=74 (%)	Total admission n=182 (%)	P-value
Age (years)	Mean (SD)	58.12 (10.4)	57.77 (11.88)	57.97 (10.99)	0.833
Gender n (%)	Male	84 (77.78)	57 (77.03)	141 (77.47)	0.905
	Female	24 (22.22)	17 (22.97)	41 (22.53)	
Ethnicity n (%)	Black	53 (49.07)	35 (47.3)	88 (48.35)	0.387
	Asian	23 (31.30)	11 (14.86)	34 (18.69)	
	Caucasian	32 (29.63)	28 (37.84)	60 (32.97)	
Presenting symptoms	Chest pain	101 (93.52)	66 (89.19)	167 (91.76)	0.297
	Other	7 (6.48)	8 (10.81)	15 (8.24)	
Length of stay (days)	Median (IQR)	3 (2-5)	4 (3-7)	3 (2-5)	0.016
**MEDICATIONS n (%)**					
Beta blockers		70 (64.81)	58 (79.38)	128 (70.33)	0.049
ACE-I		53 (49.53)	44 (59.46)	97 (53.59)	0.188
DAPT (Aspirin and Clopidogrel)		106 (98.15)	72 (97.3)	178 (97.8)	0.701
Statins		97 (89.81)	74 (100)	171 (93.96)	0.005
Nitrates		21 (19.44)	3 (4.05)	24 (13.19)	0.003
Mineralocorticoid receptor antagonists		11 (10.19)	14 (18.92)	25 (13.74)	0.093
Hypoglycemic drugs		15 (13.89)	16 (21.62)	31 (17.03)	0.173
Anticoagulants		4 (3.7)	6 (8.11)	10 (5.49)	0.2
Furosemide		3 (2.78)	7 (9.46)	10 (5.49)	0.052
Anti-arrhythmic drugs		4 (3.7)	0	4 (2.2)	0.094
Calcium channel blockers		3 (2.78)	3 (4.05)	6 (3.3)	0.636
**RISK FACTORS n (%)**					
Hypertension		70 (64.81)	47 (63.10)	117 (64.29)	0.857
Diabetes mellitus		46 (42.59)	26 (35.14)	72 (39.56)	0.312
Dyslipidemia		38 (35.19)	49 (66.22)	87 (47.80)	<0.001
Smoking		64 (59.26)	48 (64.86)	112 (61.54)	0.445
Family history of CAD		2 (1.85)	12 (16.22)	14 (7.69)	<0.001
Previous IHD		36 (33.33)	16 (21.62)	52 (28.57)	0.086
BMI >25kg/m^2^		9 (8.33)	12 (16.22)	21 (11.54)	0.102

Abbreviations: ACE-I: angiotensin converting enzyme inhibitor, BMI: body mass index, CAD: coronary artery disease, DAPT: dual antiplatelet therapy, IHD: ischemic heart disease, IQR: interquartile range, SD: standard deviation.

**Figure 2:**
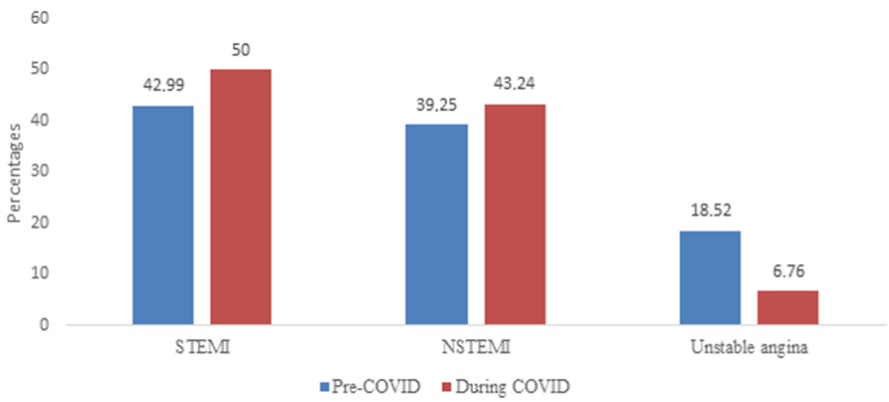
graphic presentation of patients admitted for STEMI, NSTEMI and unstable angina

Table 2 and Figure 2 summarize the specific diagnosis for which patients were admitted. STEMI accounted for the bulk of admissions in both groups, followed by NSTEMI, however, there was no statistically significant difference between STEMI and NSTEMI admissions between the two periods. More patients with UA were admitted during the pre-COVID period (18.52% vs 6.76%, P=0.013).

**Table 2:** summary of specific acute coronary syndrome diagnoses among patients admitted in the pre-COVID-19 and COVID-19 periods

Variable	Categories	Total Admissions n (%)	Pre-COVID Admissions n (%)	During COVID Admissions n (%)	Percentage change (difference) in admissions (95% CI)	P-value
ACS	STEMI	83 (45.86)	46 (42.99)	37 (50)	7% (2.1 - 6.7%)	0.321
	NSTEMI	74 (40.88)	42 (39.25)	32 (43.24)	4% (-9.6 - 17.6%)	0.5652
	UA	25 (13.74)	20 (18.52)	5 (6.76)	-12% (-21.2 - 2.8%)	0.013
STEMI	Inferior	34 (40.96)	17 (36.96)	17 (45.95)	9% (-4.6 - 22.6%)	1.29
	Anterior	49 (59.04)	29 (63.04)	20 (54.05)	-9% (-22.6 - 4.6%)	0.196

ACS: Acute coronary syndrome, CI: confidence interval, non-ST-elevation myocardial infarction, STEMI: ST-elevation myocardial infarction. UA: unstable angina.

**Figure 3:**
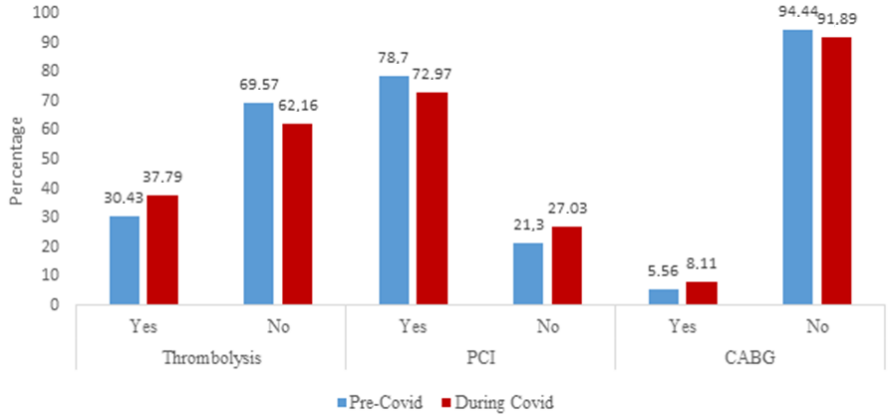
the distribution of patients who received thrombolysis for STEMI and those who underwent PCI and referral for CABG for ACS during pre-COVID-19 and COVID-19 periods

**Management of patients with ACS during pre-COVID-19 and COVID-19 periods:** this information is summarized in Figure 3. Only a third of STEMI patients received thrombolytic therapy during both periods and there was no difference noted in the two groups (P=0.478). A total of 139 PCI procedures were performed during the study period. There was no statistically significant difference in the vascular (coronary artery) territories affected for the patients who presented with STEMI. Concurrent with overseas findings, more PCI procedures were done during the pre-COVID-19 period [85 (78.70%)] compared to the COVID-19 period [54 (72.97%)], however, this did not reach statistical significance (P =0.371) in our study. There was no difference in the number of patients who underwent coronary artery bypass grafting (CABG) (P =0.495).

**Complications related to ACS admissions during pre-COVID-19 and COVID-19 periods:** Table 3 depicts complications related to ACS observed during the study period. The only significant between-group difference was that of a higher incidence of arrhythmias in patients admitted during the pre-COVID-19 period (P= 0.011). Other complications that had a trend towards statistical significance included heart failure and atrioventricular block. Six in-hospital mortalities were observed. Five patients died in the pre-COVID-19 period and one patient during the COVID-19 period.

**Table 3:** complications encountered in acute coronary syndrome patients during the pre-COVID-19 and COVID-19 periods

Variable	Pre-COVID-19 Admissions n (%)	COVID-19 admissions n (%)	Total Admissions n (%)	P-value
**Complications**	43 (38.81)	38 (51.35)	81 (44.51)	0.124
HFrEF	37 (34.26)	36 (48.65)	73 (40.11)	0.052
Cardiogenic shock	1 (0.93)	0	1 (0.55)	0.407
Arrhythmias	9 (8.33)	0	9 (4.95)	0.011
Left ventricular clot	2 (1.85)	1 (1.35)	3 (1.65)	0.794
Cardiac Arrest	2 (1.85)	1 (1.35)	3 (1.65)	0.794
Atrioventricular blocks	1 (0.93)	4 (5.41)	5 (2.75)	0.069
Critical limb ischemia	0	2 (2.7)	2 (1.1)	0.086
Left ventricular aneurysm	0	1 (1.39)	1 (0.56)	0.219

HFrEF- Heart failure with reduced ejection fraction

## Discussion

This is the first South African study to assess the impact of the COVID-19 pandemic at a public tertiary-level hospital. The study corroborates the anticipated overall reduction in ACS admissions. However, there was no difference in the rates of thrombolysis and the number of PCI and CABG procedures during the COVID-19 pandemic compared to the pre-COVID-19 period.

Similar to several international studies that showed a significant reduction in ACS admissions [6-16], our study confirmed the same. Fear of contracting COVID-19, together with the lockdown measures instituted by countries to limit the rapid spread of COVID-19, have been shown to contribute to this finding [11,20]. Other contributory factors include: 1) reduced physical activity and exertion during lockdown periods which in itself reduces myocardial oxygen demand and therefore the incidence of MI [21], 2) patients were less likely to seek medical care out of concern of an overwhelmed, resource-limited South African medical system, 3) better health practices and 4) adherence to treatment during the confines of lockdown

Moreover, we found that the number of admissions for UA decreased by 75% during the COVID-19 period. This finding was also highlighted among patients attending South African private hospitals. [17] A meta-analysis which included forty studies, concluded a 48.1% reduction in admission rates for UA during the COVID-19 pandemic (Helal *et al*.) [22]. This finding was attributed to less severe symptoms associated with UA as opposed to STEMI/NSTEMI. Thus, patients likely tended to wait instead of seeking urgent medical attention. This is particularly relevant in the South African context, where this behaviour is not uncommon even under normal circumstances due to the poor socioeconomic status of the patients compounded by the lack of access to public transport [23,24]. Other factors such as underestimation/ misinterpretation of symptoms may have also played a role [11].

It is important to note that only one-third of STEMI patients received fibrinolysis in both periods. This finding is akin to the study by Meel *et al*. and the larger Acute Coronary Events-a Multinational Survey of Current Management Strategies (ACCESS) registry study conducted approximately a decade ago in the pre-COVID-19 [25,26]. It again highlights the failing South African healthcare system and an inability to implement measures to improve the care of STEMI patients, despite attempts to establish strategies and networks in South Africa for the care of these patients [27]. The findings of the current study may be related to a combination of factors such as: 1) poorly educated patients with ignorance regarding ACS symptoms and therefore late presentations beyond the thrombolysis period; 2) poor STEMI activation systems [28] and/or 3) increase in under- and misdiagnosis during the pandemic due to fear amongst healthcare workers of contracting COVID-19 infection; [22] 4) the growing number of broadcasts from national authorities emphasizing chest discomfort and dyspnea as the dominant symptoms of COVID-19 may have been misleading for many patients with ACS [22] and 5) a strained healthcare system coupled with restructuring to accommodate acutely ill COVID-19 patients may have accounted for the misdiagnoses and inappropriate down-referral of ACS patients. We did not document the time to thrombolysis in this study during the two periods. Based on prior data, a minority of patients in South African hospitals receive fibrinolytic therapy within the optimal one- to - six-hour window period due to previously reported factors [25,27]. This translates into increased morbidity and mortality in these patients.

There was no difference in PCI procedures or CABG referral during the pre-COVID-19 period compared to the COVID-19 period. This was an interesting finding. As per initial data from China and Italy [29], we advocated the use of thrombolysis over PCI in patients with ACS during the COVID-19 period to the avoid risk of COVID-19 infection to operators in our cardiology unit. Despite postponing elective procedures at CHBAH, advocating for the use of thrombolysis, awaiting COVID swab results before PCI, and discharging low-risk patients with ACS, no difference in the rates of PCI was noted during the two periods. This lack of difference in PCI during the two periods again is likely a result of high volumes of ACS referrals to CHBAH that nullified the effect of the pandemic on overall PCI procedures. This finding is similar to other large-volume referral centres in the world [30].

There was a trend toward higher admissions secondary to ACS-related HFrEF and higher atrioventricular block in the COVID-19 period. This was likely due to delayed presentations and lack of timeous PCI or thrombolysis during this period. Arrhythmias in ACS generally occur during the first few hours following the onset of symptoms [31]. As patients likely presented late during the pandemic, it may explain why arrhythmias were less frequently observed during this period, resulting in a significant difference between the two groups. The increased incidence of complications related to delayed presentation of ACS during the COVID-19 pandemic has been observed and reported previously in Chinese and Italian studies [28]. Nevertheless, in the South African context, these findings are of grave concern as it is likely due to overburdened healthcare facilities due to repeated hospitalizations of such patients with heart failure and other related complications. This has a direct effect on the costs incurred by these facilities. It has been shown that the cost of a heart failure-related admission can be between ZAR 135 to ZAR 733 per night at CHBAH [32]. Additionally, the high rates of complications post-MI impair the quality of life of these patients and place economic and psychological burdens on patients and their families [33,34]. These problems must be anticipated, and plans need to be put in place to limit the effects of the aforementioned issues during the post-COVID period.

We also noted a more aggressive usage of medical therapy in the form of beta blockers, statins, nitrates, and furosemide during the COVID-19 period compared to the pre-COVID-19 period. The current literature suggests no difference in the medical management of ACS patients during the COVID-19 period, except for an emphasis on anticoagulation in patients who are confirmed COVID-19 positive [29]. The patients admitted to CCU during the COVID-19 period were sicker with higher risk features and complications such as heart failure. This would have led to a significant difference in the usage of prior medication by the clinician in this study. This finding was in contrast to the multicenter study conducted in Israel by Fardman *et al*. [35], whereby no difference in the distribution of medication was noted in the COVID-19 and non-COVID periods amongst STEMI patients. A greater prevalence of dyslipidemia and a strong family history of CAD were in our view, coincidental findings that may not have contributed to the higher complication rates.

Furthermore, this study found that patients admitted during the COVID-19 period had a longer hospital stay compared to pre-COVID-19 with a median of 4 days. This is in contrast to a French study by Matsushita *et al*. and an English study by Kwok *et al*. where hospital stay was shorter [36,37]. This discrepancy in our hospital is explained by the fact that patients required a negative COVID-19 polymerase chain reaction (PCR) test before admittance to CCU. This was further aggravated by laboratory delays and the need for repeat testing in patients who were transferred from outlying hospitals. The longer hospital stay has cost implications and also increases a patient's risk of acquiring COVID-19 during the hospitalization period.

**Limitations:** the retrospective nature of the study suffers from common limitations inherent in such a study design. Most other studies compared the time during lockdown measures to a similar period in the previous year. We were unable to do the same. However, as CHBAH attends to high volumes of patients daily, we did not believe that this would have a substantial effect on the results of this study. In addition, the paucity of data for patients who died in CCU, or those with ACS who were not admitted to CCU greatly impacts the cohort size as well as deductions that can be made about hard endpoints such as morbidity and mortality. All patients admitted to CCU were COVID negative and therefore patients who were COVID-19 positive with concurrent ACS were inevitably excluded from the analysis. Further, it will be difficult to ascertain the true number of ACS admissions outside of CCU that was admitted to the COVID ward due to the high number of confounders for troponin leakage in the setting of concurrent COVID-19 infection, as lack of meticulous record keeping and missing data make it difficult to access such information in a large volume centre. In a recent study by Meel *et al*. [38], a prevalence of 6.5% for ACS in patients with COVID-19 was noted in a study conducted in late 2020-2021 at CHBAH and we suspect a similar trend may have occurred in the early part of the pandemic. Further, it must be noted that the intent of the study was not to study the effect/pathophysiology of COVID-19 and concurrent ACS but rather the influence of the pandemic on ACS admissions and management irrespective of their COVID-19 status. Lastly, this study focused on a single centre and therefore the results cannot be extrapolated to assess the effect of the COVID-19 pandemic at the district, provincial and national levels and other countries.

## Conclusion

There was an overall difference in ACS admissions during the pre-COVID and COVID-19 periods, however no difference in STEMI and NSTEMI admissions during both periods in the current study. Fewer cases of unstable angina were seen during the COVID-19 period. During both periods only a third of the patients with STEMI received fibrinolysis and no difference in PCI or CABG was noted. There were no differences in complications observed between the two periods, except for a higher rate of arrhythmias noted in the pre-COVID-19 period. The COVID-19 pandemic has had an unprecedented impact on cardiology services worldwide and has had an even greater impact on the already under-resourced developing world as suggested by mostly anecdotal reports in the absence of formal large studies. We hope that this study, despite its limitations, has shed light on the impact of COVID-19 on ACS admissions at a tertiary hospital in South Africa. Below, we outline some brief recommendations regarding the management of ACS in the era of the COVID-19 pandemic in the South African context.

**Recommendations:** education of the general population through social media platforms about early recognition of ACS symptoms and immediate referral to emergency departments is of utmost importance. The dangers of MI-related complications that may result from delays in seeking medical help must be emphasized. Anxiety and fears associated with the acquisition of COVID-19 infection at health institutions must be addressed and patients must be reassured regarding the preventative protocols that are implemented at most hospitals to prevent transmission of infection.

All patients with ACS requiring emergency care and who are not candidates for thrombolytic therapy or who require rescue PCI must be assumed to be COVID-positive and time should not be wasted whilst awaiting results of the COVID swab. The catheterization laboratory personnel must be trained in the management of these patients and should have undergone appropriate personal protective equipment (PPE) training. Only the minimum number of staff needed for the case must be present in the catheterization laboratory. The case must be performed by an experienced operator to minimize prolonged exposure to a patient with possible COVID-19 infection. The catheterization laboratory must be sanitized upon completion of the procedure. A dedicated isolation area in the hospital must be reserved for ACS patients where they can be monitored post-procedure whilst awaiting the results of the COVID-19 test.

### What is known about this topic

- *There was a significant reduction worldwide in the number of hospitalizations of patients with ACS and those requiring PCI during the COVID-19 pandemic;*
- *The stringent measures put in place to contain the pandemic not only threatened the economy but also posed a threat to public health and the care of non-COVID-related cases*.

### What this study adds

- *This study adds further weight on global data that COVID-19 did affect admissions and care of ACS patients;*
- *It gives additional data to the scarce pool of literature emanating from Africa with regards to the care of patients with ACS and recommendations pertaining to the management during the COVID-19 pandemic*.

## References

[1] Wu Z, McGoogan JM (2020). Characteristics of and important lessons from the coronavirus disease 2019(COVID-19) outbreak in China. Summary of a report of 72314 cases from the Chinese Centre for Disease Control and Prevention. JAMA.

[2] World Health Organization (WHO) Report of the WHO-China Joint Mission on Coronavirus Disease 2019 (COVID-19).

[3] Wilder-Smith A, Bar-Yam Y, Fisher D (2020). Lockdown to contain COVID-19 is a window of opportunity to prevent the second wave. J Travel Med.

[4] South Africa African Government Regulations and Guidelines - Coronavirus COVID-19.

[5] Pessoa-Amorim G, Camm CF, Gajendragadkar P, De Maria GL, Arsac C, Laroche C (2020). Admission of patients with STEMI since the outbreak of the COVID-19 pandemic: a survey by the European Society of Cardiology. Eur Heart J Qual Care Clin Outcomes.

[6] De Rosa S, Spaccarotella C, Basso C, Calabrò MP, Curcio A, Filardi PP (2020). Reduction of hospitalization for myocardial infarction in Italy in the COVID-19 era. Eur Heart J.

[7] Metzler B, Siostrzonek P, Binder RK, Reinstadler SJ (2020). Decline acute coronary syndrome admissions in Austria since the outbreak of covid-19: the pandemic response causes cardiac collateral damage. Eur Heart J.

[8] Rodrigues-Leor O, Cid-Alvarez B, Ojeda S, Martín-Moreiras J, Rumoroso JR, López-Palop R (2020). Impact of the covid-19 pandemic on interventional cardiology activity in Spain. REC Interv Cardiol.

[9] Mesnier J, Cottin Y, Coste P, Ferrari E, Schiele F, Lemesle G (2020). Hospital admissions for acute myocardial infarction before and after lockdown according to regional prevalence of COVID-19 and patients profile in France: a registry study. Lancet Public Health.

[10] Schmitz T, Meisinger C, Kirchberger I, Thilo C, Amann U, Baumeister SE (2021). Impact of COVID-19 pandemic lockdown on myocardial infarction care. Eur J Epidemiol.

[11] Tam CF, Cheung KS, Lam S, Wong A, Yung A, Sze M (2020). Impact of coronavirus disease 2019 (COVID-19) outbreak on outcome of myocardial infarction in Hong Kong, China. Circ Cardiovasc Qual Outcomes.

[12] Shams P, Faheem O, Adnan G, Ali J, Khan M (2020). Cardiovascular Interventions in Face of Covid-19 Pandemic in a Low to Middle Income Country of South Asia Region-A Pre and Post Covid Era Comparison. Circulation.

[13] Morishita T, Takada D, Shin J-ho, Higuchi T, Kunisawa S, Imanaka Y (2022). Trends, Treatment Approaches, and In-Hospital Mortality for Acute Coronary Syndromes in Japan During the Coronavirus Disease 2019 Pandemic. J Atheroscler Thromb.

[14] Braiteh N, Rehman WU, Alom M, Skovira V, Breiteh N, Rehman I (2020). Decrease in acute coronary syndrome presentations during the COVID-19 pandemic in upstate New York. Am Heart J.

[15] Garcia S, Albaghdadi MS, Meraj PM, Schmidt C, Garberich R, Jaffer FA (2020). Reduction in ST-segment Elevation Cardiac Catheterization Laboratory Activations in the United States during COVID-19 Pandemic. J Am Coll Cardiol.

[16] Mayol J, Artucio C, Batista I, Puentes A, Villegas J, Quizpe R (2020). An international survey in Latin America on the practice interventional cardiology during the COVID-19 pandemic, with a particular focus on myocardial infarction. Neth Heart J.

[17] Delport R, Vachiat A, Snyders A, Kettles D, Weich H (2020). Decline in acute coronary syndrome hospitalization rates during COVID-19 lockdown in private hospitals in South Africa. SAHeart.

[18] Anderson JL, Morrow DA (2017). Acute myocardial infarction. N Engl J Med.

[19] Thygesen K, Alpert JS, Jaffe AS, Chaitman BR, Bax JJ, Morrow DA (2018). Fourth universal definition of myocardial infarction (2018). J Am Coll Cardiol.

[20] Huynh K (2020). Reduced hospital admissions for ACS-more collateral damage from COVID-19. Nat Rev Cardiol.

[21] Arnold SV, Smolderen KG, Buchanan DM, Li Y, Spertus JA (2012). Perceived stress in myocardial infarction: long-term mortality and health status outcomes. J Am Coll Cardiol.

[22] Helal A, Shahin L, Abdelsalam M, Ibrahim M (2021). Global effect of COVID-19 pandemic on the rate of acute coronary syndrome admissions: a comprehensive review of published literature. Open Heart.

[23] Organisation for Economic Co-operation and Development World Health Organization DAC Guidelines and Reference Series-Poverty and Health.

[24] Ngubane L The state of public transport in South Africa.

[25] Meel R, Goncalves R (2015). Time to fibrinolytics for acute myocardial infarction: Reasons for delay at Steve Biko Academic Hospital, Pretoria, South Africa. S Afr Med J.

[26] Schamroth C; ACCESS South Africa investigators (2012). Management of acute coronary syndrome in South Africa: insights from the ACCESS (Acute Coronary Events-a Multinational Survey of Current Management Strategies) registry. Cardiovasc J Afr.

[27] Snyders A, Delport R (2015). Referral pathways for reperfusion of STEMI-developing strategies for appropriate intervention: the SA heart STEMI early intervention project. SA Heart.

[28] Cameli M, Pastore MC, Mandoli GE, D'Ascenzi F, Focardi M, Biagioni G (2021). COVID-19 and Acute Coronary Syndromes: Current Data and Future Implications. Front Cardiovasc Med.

[29] Nijjer SS, Petraco R, Sen S (2020). Optimal management of acute coronary syndromes in the era of COVID-19. Heart.

[30] Toner L, Koshy AN, Hamilton GW, Clark D, Farouque O, Yudi MB (2020). Acute coronary syndromes undergoing percutaneous coronary intervention in the COVID-19 era: comparable case volumes but delayed symptom onset to hospital presentation. Eur Heart J Qual Care Clin Outcomes.

[31] O´Doherty M, Tayler DI, Quinn E, Vincent R, Chamberlain DA (1983). Five hundred patients with myocardial infarction monitored within one hour of symptoms. Br Med J (Clin Res Ed).

[32] Revision of uniform patient fee schedule relating to the (2019). Classification of and fees payable by patients at provincial. Hospitals.

[33] Mollon L, Bhattacharjee S (2017). Health related quality of life among myocardial infarction survivors in the United States: a propensity score matched analysis. Health Qual Life Outcomes.

[34] Petersen I, Bhana A, Folb N, Thornicroft G, Zani B, Selohilwe O (2018). Collaborative care for the detection and management of depression among adults with hypertension in South Africa: study protocol for the PRIME-SA randomised controlled trial. Trials.

[35] Fardman A, Zahger D, Orvin K, Oren D, Kofman N, Mohsen J (2021). Acute myocardial infarction in the Covid-19 era: Incidence, clinical characteristics and in-hospital outcomes-A multicentre registry. Plos One.

[36] Matsushita K, Hess S, Marchandot B, Sato C, Truong DP, Kim NT (2021). Clinical features of patients with acute coronary syndrome during the COVID-19 pandemic. J Thromb Thrombolysis.

[37] Kwok CS, Gale CP, Kinnaird T, Curzen N, Ludman P, Kontopantelis E (2020). Impact of COVID-19 on percutaneous coronary intervention for ST-elevation myocardial infarction. Heart.

[38] Meel R, Van Blydenstein SA (2021). Demographic, clinical, electrocardiographic and echocardiographic characteristics of patients hospitalized with COVID-19 and cardiac disease at tertiary hospital, South Africa. Cardiovasc Diagn Ther.

